# Microstructural Characterization and Corrosion Behavior of Similar and Dissimilar Welded Advanced High-Strength Steels (AHSS) by Rotary Friction Welding

**DOI:** 10.3390/ma17040918

**Published:** 2024-02-16

**Authors:** Antonio Enrique Salas Reyes, Gabriel Ángel Lara Rodriguez, Jesús Rafael González Parra, Víctor Hugo Mercado Lemus

**Affiliations:** 1 Departamento de Ingeniería Metalúrgica, Facultad de Química, UNAM, Cuidad de México 04510, Mexico; rafael.parra@comunidad.unam.mx; 2 Instituto de Investigaciones en Materiales, UNAM, Ciudad de México 04510, Mexico; larag@unam.mx; 3 Centro de Ingeniería de Superficies y Acabados, Facultad de Ingeniería, UNAM, Ciudad de México 04510, Mexico; 4 CONACYT-Corporación Mexicana de Investigación en Materiales, San Luis Potosí 78395, Mexico; victor.mercado@ciateq.mx

**Keywords:** rotary friction welding (RFW), similar and dissimilar welding, AHSS, TWIP steel, complex-phase steel, quenching and partitioning steel, corrosion behavior

## Abstract

Advanced high-strength steels (AHSSs) are designed for meeting strict requirements, especially in the automotive industry, as a means to directly influence the reduction in the carbon footprint. As rotary friction welding (RFW) has many important advantages over other welding technologies, it plays an important role in the automotive sector. On the above basis, in this work, combinations of the first (complex phase (CP)), second (TWIP (TW)), and third (quenched and partitioned (Q&P)) generations of similar and dissimilar high-alloyed advanced steels have been joined by the RFW process. Having a specific microstructure, rods of CP/CP, Q&P/Q&P, CP/TW, and Q&P/TW steels were welded by employing a homemade adaptation machine under fixed parameters. Microstructural characterization has allowed us to corroborate the metallic bonding of all the tested advanced steels and to identify the different zones formed after welding. Results indicate that the welding zone widens in the center of the workpiece, and under the current friction action, the intermixing region shows the redistribution of solute elements, mostly in the dissimilarly welded steels. Furthermore, because of their complex chemistry and the different mechanical properties of the used steels, dissimilarly welded steels present the most noticeable differences in hardness. The TWIP steel has the lower hardness values, whilst the CP and Q&P steels have the higher ones. As a direct effect of the viscoplastic behavior of the steels established by the thermomechanical processing, interlayers and oxidation products were identified, as well as some typical RFW defects. The electrochemical response of the welded steels has shown that the compositional and microstructural condition mostly affect the corrosion trend. This means that the dissimilarly welded steels are more susceptible to corrosion, especially at the TWIP–steel interface, which is attributed to the energy that is stored in the distorted microstructure of each steel plate as a consequence of the thermomechanical processing during RFW.

## 1. Introduction

Solid-state welding is a diffusion-controlled process. The metallic solid-state welding processes consider the technologies that produce the mechanical coalescence of the surfaces of the materials to be joined at temperatures below their melting point. In the case of rotary friction welding (RFW), the heat generated by the friction between two surfaces, or two workpieces, is used to obtain the metallic union of similar and dissimilar alloys. The nature of RFW is essentially governed by the atomic coalescence of two solid surfaces free of dirt, categorized in the sequence of three metallurgical events, called rigid and severe plastic deformation, and mass transport induced by the diffusion between interfaces [1]. Thus, the joining is generated by atomic diffusion, followed by dynamic recrystallization and/or grain growth along the welded interface.

In essence, metallic union is induced by the mechanical combination between the friction and axial forces that basically simulates a metallurgical forge. The plastic deformation generates materials to flux and forms a flash collar [2]. Obviously, flash-collar formation trims the total length of both workpieces.

Likewise, there are many advantages of using RFW: there is no external source used for heating the materials nor for the filler material, there is low environmental pollution, and it is cost-effective. Most of the time, the metallic union has a high quality and, obviously, the final joint presents outstanding mechanical properties [3].

The RFW process is very efficient in terms of productivity, allowing a high number of joints to be obtained in a short time. It is also important to point out that this process makes it possible to weld even those alloys considered difficult to weld using the more conventional melting processes. However, although the RFW technology (i.e., the equipment and instrumentation control) is still under development, its applicability is limited by the workpiece geometry, which practically uses symmetric cylindrical shapes [4].

RFW was initially developed by the extinct Soviet Union [5], where the first experiments date back to 1956 and resulted in several patents. In addition, major American companies, such as Caterpillar, Rockwell International, and the American Manufacturing Foundry, developed equipment and machinery for this process [5]. From that time until today, many research works have been carried out, focusing their efforts on understanding both the effect of the welding parameters and the involved phenomena that take place as a consequence of the thermomechanical cycle applied to achieve the welding and its influence on the mechanical properties of the joined materials. Likewise, in recent decades, the automotive industry has undoubtedly been at the forefront of research, developing and applying new materials (e.g., metallic, polymeric, ceramic, and composite materials) that meet new environmental regulations worldwide. This means that it is necessary to manufacture light cars with the use of the highest quality technology that contributes to the reduction of gas emissions and, at the same time, ensures a high level of car safety in the event of crashes and collisions, thus increasing profitability in accordance with customer satisfaction.

For this reason, one of the most innovative solutions has been the use of advanced high-strength steels (AHSSs). These steels offer an excellent mechanical strength while maintaining good formability throughout all manufacturing processes. Their implementation has promoted the reduction of thinner cross-sectional pieces and, consequently, the reduction of the total mass of vehicles [6].

Several studies have been reported in the specialized literature focusing on the relationship between the RFW process parameters–microstructure–mechanical properties of various steel grades, including similar and dissimilar welds. Durkenton [7] was one of the first to compare various friction welding techniques and concluded that, for carbon steels welded by simple rotation, the quality of the joint is a function of the content population of nonmetallic inclusions, since they cause embrittlement. On the other hand, the shape of the flash collar is dependent on the rotational speed, the preload, and the forging load [8]. Rotational speeds ranging from 700 to 2000 rpm have been evaluated [9]. Even empirical and numerical models have been developed to estimate the scope and optimization of this type of technology [10,11,12,13]. Also, novel microstructural characterization techniques to analyze the resulting microstructure in the different formed zones (i.e., the thermomechanical-affected zone (TMAZ), the thermal-affected zone (TAZ), and the welded zone (WZ)) after RFW have been applied [14].

Naturally, the metallurgical linkage between each of the surface features in RFW presents challenges in the joining process. In the case of similar welding, there are no major complications in obtaining the metallic union. However, the fact of welding dissimilar materials makes the metal joint difficult due to the differences in their melting points, as well as their thermal and conductivity behavior, and can consequently promote the materials being welded to form harmful precipitation particles between them [15]. Getting away from the interface union, in the case of those carbon steels that experiment with phase transformation, they can show two microstructures, namely, TMAZ and HAZ. Near the interface zone, finer ferrite and pearlite grains can be found in the TMAZ, produced principally by a recovery and recrystallization of metallurgical mechanisms. However, the HAZ is formed due to the larger size, containing coarser grains. On the contrary, the severe plastic deformation achieved during RFW has allowed us to build the WZ. Also, in dissimilar welds, it can be found in some layers in the WZ, which form as a consequence of the applied axial force during the intermixing of both materials [16].

However, it has been pointed out that there are no reported scientific works yet that have dealt with RFW technology in AHSSs, neither in similar nor in dissimilar welds. On the contrary, due several body-in-white vehicle parts manufactured by AHSS using diverse welding technologies, such as resistance spot welding (RSW), laser welding (LW), high-power laser beam welding (HPLBW), and hybrid welding (HW), have been revised in detail in [17], although other welding processes are also found for AHSSs, such as friction stirring welding (FSW) [18,19] and friction stir spot welding (FSSW) [20]. The most recent studies agree on the usefulness of the application of solid-state welding techniques in advanced steels because, in a certain sense, they can limit the damage to the microstructure caused by the temperature.

At this point, it is important to mention that steel is the most widely used material in the automotive industry due to its flexibility and enormous economic and social benefits. Thus, the automotive industry is taking steps to replace various materials, including diverse carbon steel grades, by promoting the use of advanced materials with superior strength, formability, and durability to ensure increased structural integrity, at a lower cost, in the final products [21,22]. So, there are a number of different ways to classify automotive steels. One is a metallurgical designation that provides some process information. Steel strength is a second classification method important to part designers. High-strength, low-alloy (HSLA) steels, also known as microalloyed (MA) steels, have a microstructure consisting of fine-grained ferrite that can be strengthened with carbon and/or nitrogen precipitates of titanium, vanadium, or niobium. HSLA steels can be formed successfully when users know the limitations of the higher-strength, lower-formability trade-off [23]. Contrarily, AHSSs are low-carbon steel grades designed in the last 30 years as an answer to the contemporary requirements of the automotive industry for lighter, more crash-resistant, and fuel-efficient vehicles. They combine excellent weldability, formability, galvanizing ability, and crash resistance with a high strength, toughness, and fatigue resistance [24]. Furthermore, the principal difference between conventional HSLA steels and AHSSs is in their microstructure. Therefore, the basic idea of the lightweight design in the automotive industry, by increasing the usage of AHSS, is to create high-performance structures with a minimum weight while meeting the essential requirements, such as technical requirements, safety, and the reasonable use of energy technologies [25].

Hence, the rotary friction welding technology can be one of the reasons to multiply the applications of the AHSS in the manufacture of other types of automotive components (i.e., nonbody-in-white car-body frames), such as camshafts, connecting rods, gears and homokinetic shafts, ball joints, etc. For this reason, it is very relevant to conduct a study focused on the metallurgical capacity of welding similar and dissimilar first generation (complex phase (CP)), second generation (TWIP (TW)), and third generation (quenched and partitioned (Q&P)) combinations of high-alloyed advanced steels by the RFW technology. And, since these advanced steels can present complex chemistries in the welded zone (WZ) due to the metallurgical effects involved after RFW, corrosion is expected to occur, which is also the concern for measuring their corrosion resistance in the present work.

## 2. Materials and Methods

### 2.1. Treatment of Advanced Steels before Welding (Microstructure Conditioning)

**Figure 1:**
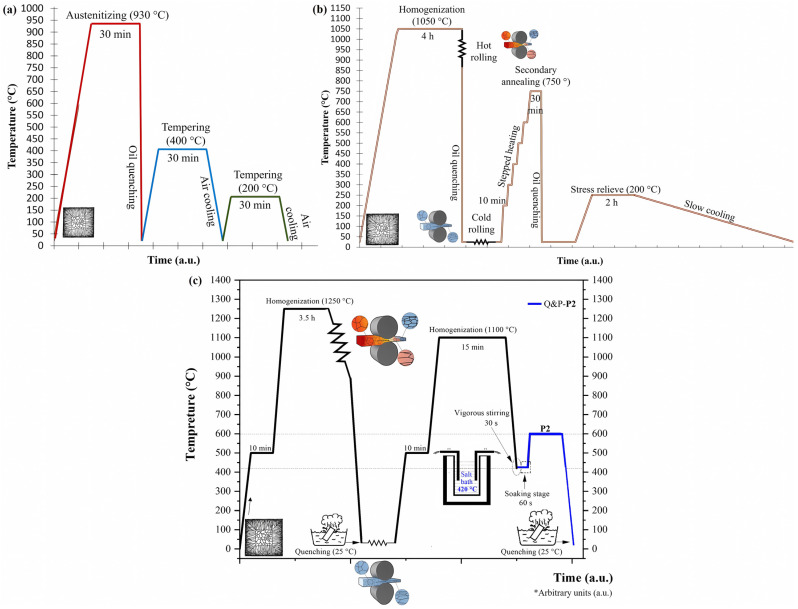
Thermal cycle for the microstructure conditioning of the 1st, 2nd, and 3rd generations of advanced steels: (**a**) CP steels, (**b**) TWIP steels, and (**c**) Q&P-P2 steels.

For carrying out the rotary friction welding tests, two complex-phase (CP) steels, identified as CP-B0 and CP-B3, two TWIP steels, identified as TW-B0 and TW-B2, and two quenched and partitioned (Q&P) steels, identified as Q&P-B0/P2 and Q&P-B3/P2, in which P2 was used for indicating the two-step partition treatment, were employed. Also, it is important to note that the CP steels were converted to Q&P steels. These advanced steels were fabricated in an open-air induction furnace employing nonpure raw materials following the steps described previously in [26,27], and their chemical compositions are shown in Table 1. The main difference between each generation of advanced steel is the boron content. On the one hand, the CP steels were quenched and tempered from their as-cast microstructure following the thermal cycle shown in Figure 1a. On the other hand, the TWIP and Q&P steels were treated as indicated by the thermomechanical, mechanical, and thermal cycles shown in Figure 1, incises b and c. Thus, following the cited thermal cycles, a final microstructure was obtained in each advanced steel (i.e., microstructure conditioning).

For each advanced steel, microstructural characterization was carried out by employing an Olympus PMG 3 optical microscope (Olympus Corporation of the Americas(OCA), Center Valley, PA, USA) and a JEOL JSC-6000 Plus Neoscope scanning electron microscope (JEOL Ltd., Tokyo, Japan). The microstructure of the CP steels was carried out using a chemical solution of LePera [28] by immersion for 2 min at room temperature. For the TWIP steels, it was revealed using Nital-10% [28] at 60 °C by immersion for 20 s. And for the Q&P steels, the microstructure was revealed by employing a modified solution of the LePera reactant [27] by immersion for 35 s at 70 °C.

### 2.2. Welding, as-Welded Metallographic Sample Preparation, and Corrosion Test Procedures

**Figure 2:**
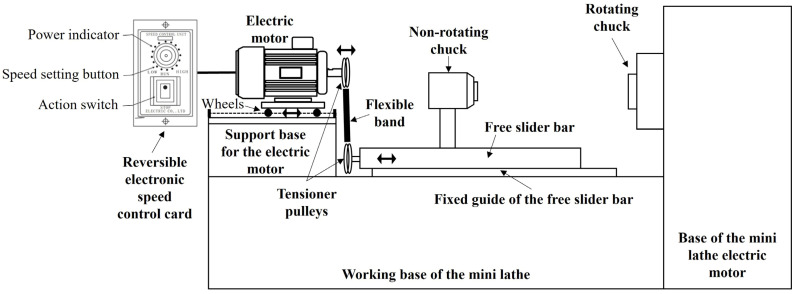
In-house mini-lathe adaptation for the rotary friction welding (RFW) tests.

Rods with a diameter of 1.3 cm and 8 cm of length were machined in the rolling direction from the plates of the different advanced steels (CP, TWIP, and Q&P) in the final microstructure, as shown in Figure 1, also referred to here as the “as-conditionated microstructure” (i.e., the base materials). Special care was paid in the transversal surface of the rods, with the aim of obtaining flat and smooth surfaces by grinding them using SiC papers (i.e., from #180 to #1000) (Fandeli, Mexico). These rods were cleaned in ethanol using an ultrasonic bath. Thus, rotary friction welding (RFW) was performed by employing an in-house mini-lathe machine, which was equipped with our own in-house-design additaments that allowed for the application of the preload and forge load. In fact, this equipment had an electric pusher connected with a free-moving rail, which was controlled by a DC motor speed electronic card (see Figure 2). So, taking into account previous calculated loads, the displacement of the rod samples performed by the mobile system was ensured using a torque wrench. With this procedure, it was possible to apply the load to achieve the metallic joints. In accordance with the above, a fixed speed of 850 rpm and a fixed preload of 1.5 MPa, as well as a fixed forging load of 4 MPa, were used. Thus, similar welding tests consisted in the joining of the CP-B0/CP-B3 steels and the Q&P-B0/Q&P-B3 steels. Additionally, dissimilar welding tests consisted in the joining of the CP-B0/TW-B2 steels and the TW-B0/Q&P-B3 steels. After welding, each welded sample was middle-cut-off in its longitudinal section. Once each sample was divided in half, one sample was used for the microstructural and mechanical characterization and the other one was used for the corrosion testing.

The microstructural characterization of the welded steels was carried out by the same above-mentioned optical microscope and scanning electron microscope. Hence, the samples were metallographically prepared using SiC papers (i.e., from #180 to #2000) and polished with diamond pastes (i.e., 3, 1, and 0.1 µm, respectively). Chemical etching was focused on revealing the thermomechanical-affected zone (TMAZ), the thermal-affected zone (TAZ), the welded zone (WZ), and the base-material zone (BMZ) at the same time. For this purpose, similar welds were firstly etched by immersion using LePera reactant for 10 s, and secondly by using Nital-10% at 60 °C and etched for 20 s, respectively. Both etching procedures signified a good manner for revealing the microstructure of the welded steels. In the case of the dissimilar welds, the etching procedure was quite complicated. However, the same above-described etching procedures for the similarly welded steels were employed. Thus, micrographs of the better material zones were obtained because, on one side of the weld, the microstructure was well-observed and, on the other side, the microstructure was over-etched.

The mechanical resistance of both the similarly and dissimilarly welded steels was estimated by Vickers microhardness measurements at a load of 0.5 kg_f_ using a Novotest TC-MCV-1A microhardness tester (NOVOTEST Ldt., Novomoskovsk, Ukraine). Accordingly, from the welding interface, 40 indentations were performed in each of the similar welds, with an indentation separation of 200 µm between them. In the case of the dissimilar welds, 80 indentations were performed in each sample, considering a separation among each indentation of 100 µm. For the similar welds, this procedure was decided due to the similarities between the materials. Contrarily, for the dissimilar welds, it was preferred to carry out more indentations (i.e., almost double than in the similar welds) to track any change in the hardness as a consequence of the thermomechanical processing imposed on the steels (i.e., the thermophysical and chemical material differences).

**Figure 3:**
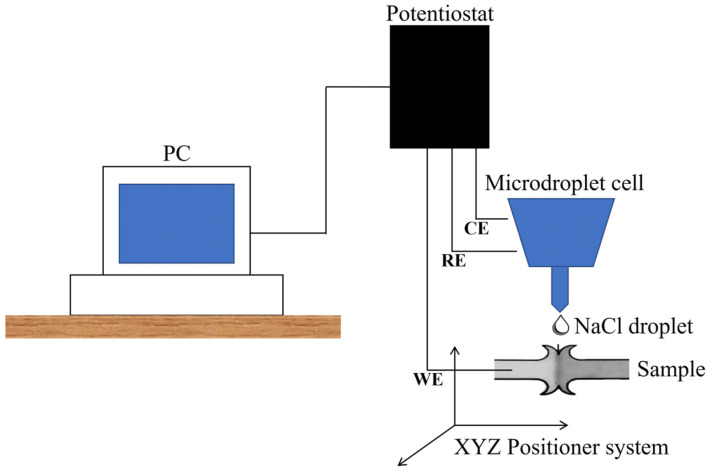
Schematic illustration for the potentiodynamic polarization measurements.

The corrosion behavior of the similarly and dissimilarly welded steels was evaluated by potentiodynamic polarization techniques using an Autolab PGSTAT 205 potentiostat (Ecochemie, The Netherlands) from a star potential of −400 mV vs OCP to a stop potential of 1000 mV vs. OCP with a current limit of 100 mA. The tests were performed using an in-house lab-made microdroplet electrochemical cell filled with a naturally aerated 3.5% NaCl solution. The electrode arrangement consisted of an Ag/AgCl reference electrode, a platinum wire as the counter-electrode, and the metal surface as the working electrode. The design of the microdroplet electrochemical cell allowed for the measurement of the response of a confined surface (approximately 1100 μm in diameter). In addition, the sample could be positioned in the XYZ to allow for the scanning of the metallic surface. For the characterization of the samples, the next procedure was followed: the microdroplet cell was positioned close to the metallic surface, wetting the surface in a confined area (≈550 μm of diameter), and, after a stabilization time, the potentiodynamic curve was obtained. Later, the microdroplet cell was displaced in the Z-axis to completely separate the electrolyte from the surface, followed by a 1500 μm displacement in the X-axis to measure the new polished surface. The potentiodynamic curves were obtained at 1 mV/s after a stabilization time of 15 min. The scheme in Figure 3 shows the arrangement used for the electrochemical characterization of the welded samples.

## 3. Results and Discussion

### 3.1. Microstructure of the Base Steels

**Figure 4:**
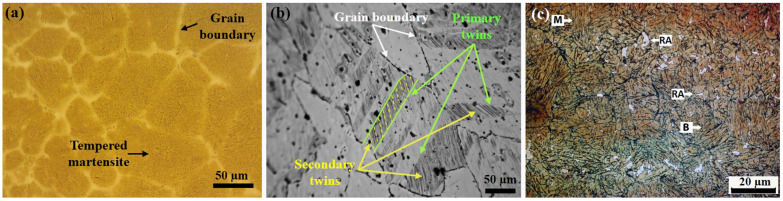
Microstructure of base metal for the welding tests: (**a**) tempered martensite in the CP steel, (**b**) deformation twins in the TWIP steel, and (**c**) multiphasic microstructure in the Q&P steel (martensite = M; bainite = B; retained austenite = RA).

Figure 4 shows the initial microstructures of the advanced steels to be welded. In the case of the CP steel (incise a), it can be seen that the heat treatment consisting of the quench and double tempering has modified the dendritic structure, which now consists of tempered martensite. It is well known that the formation of tempered martensite is achieved under low heating to overcome residual heat effects on the steel microstructure after fast cooling during quenching [29]. In the case of the TWIP steel (incise b), primary and secondary mechanical twins are observed. The effects of the annealing treatment and fast cooling have caused a dislocation pile-up, promoting the rearrangement of the deformation twins, in which secondary twins have formed in wider twins within the larger austenitic grains [30,31]. And, in the case of the Q&P steel (incise c) treated with the two-stepped (P2) heat treatment, a multiphasic microstructure consisting of a martensitic–bainitic matrix with islands of retained austenite can be seen. In particular, complex microstructures can be achieved by the Q&P treatment based on the migration of the existing carbon into the martensite structure to the soft phases, such as retained austenite, in which bainite can also favor the carbon enrichment of the retained austenite [32,33].

### 3.2. Microstructure of the Welded Steels

**Figure 5:**
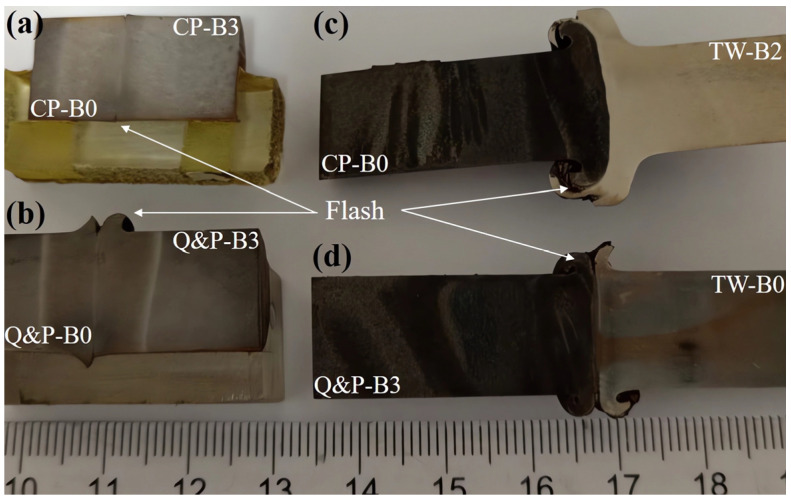
Physical appearance of etched advanced welded steels by RFW process: (**a**) the similar welding of the CP-B0/CP-B3 steels, (**b**) the similar welding of the Q&P-B0/Q&P-B3 steels, (**c**) the dissimilar welding of the TW-B2/CP-B0 steels, and (**d**) the dissimilar welding of the TW-B0/Q&P-B3 steels.

Four welded specimens were obtained after the rotary friction welding (RFW) tests. Figure 5 shows the macroscopic apparency of them along the longitudinal section at the middle of their diameter. It can be noted that, in the similarly welded steels, the flash after obtaining the metallic joint is not well-defined, as opposed to the dissimilarly welded steels, in which the flash is well-defined and has a material plastic flow orientation controlled by the interaction between the hard and soft steel, respectively. In the hard metallic materials with a closer hardness between each other and welded under a low rotational speed, the physical apparencies of the joint have a limited flow of material forming the flash [34]. This same behavior has been obtained in the present work in the similarly welded steels (i.e., the CP-B0 steel and the CP-B3 steel, and for the Q&P-B0 steel and Q&P-B3 steel). However, this behavior of the flash is not maintained in the case of the dissimilar welds (i.e., CP-B0 with TW-B2 and for the TW-B0 with Q&P-B3 steels, respectively).

**Figure 6:**
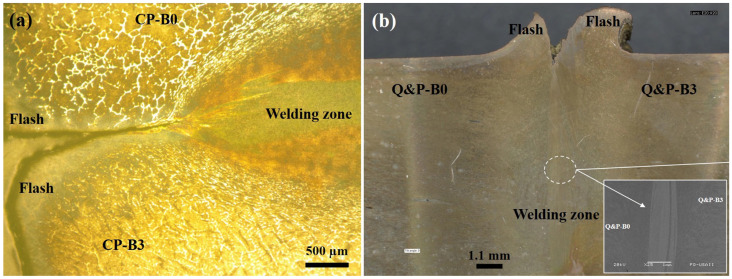
(**a**,**b**) Optical micrographs that show the widening in the welding zone for the similarly welded steels formed during the RFW tests.

**Figure 7:**
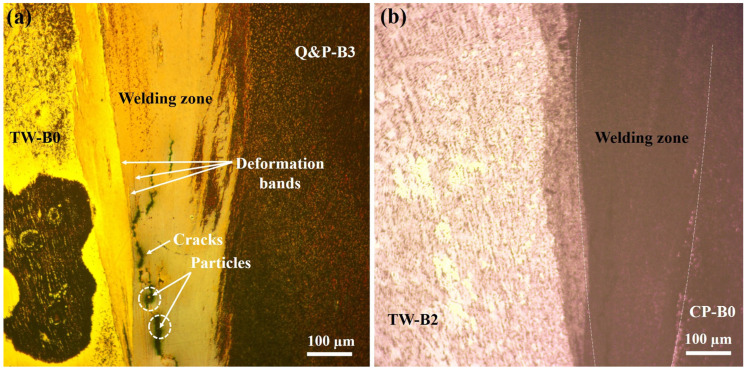
Optical micrographs that show the widening in the welding zone for the dissimilarly welded steels formed during the RFW tests: (**a**) TW-B0/Q&P-B3 and (**b**) TW-B2/CP-B0.

The weld features formed between the joining for the advanced steels are shown in Figure 6 for the similar welds and in Figure 7 for the dissimilar welds. The experimental results show that the steels have been welded satisfactorily. In the case of the similar CP welds, an SEM image has been intentionally provided to show the widening of the welding zone. It is important to note that, due to the nature of the metallic material between the dissimilarly welded steels, the revealing of their microstructures was very difficult.

Likewise, it can be seen that the material near the welding line has widened, showing a larger zone in the center of the rod and a thinner side near the surface of the rod. These welding features happened in all of the RFW tests of the similarly and dissimilarly welded steels. This welding behavior was, contrarily, different to the most common rotary friction welds, for which the flow of the material moved from the center of the rod and widened near the surface of the rod [14]. In the same manner, it can be observed that the welding zone is enclosed by a larger zone. So, the welding zone might be formed by the mixture (also called the intermixing region) of the two materials, whilst the larger zone might indicate the thermomechanical-affected zone. Getting away from this larger zone, it can be seen that the material flow provides the form of the main welding procedure, mainly in the heat-affected zone.

**Figure 8:**
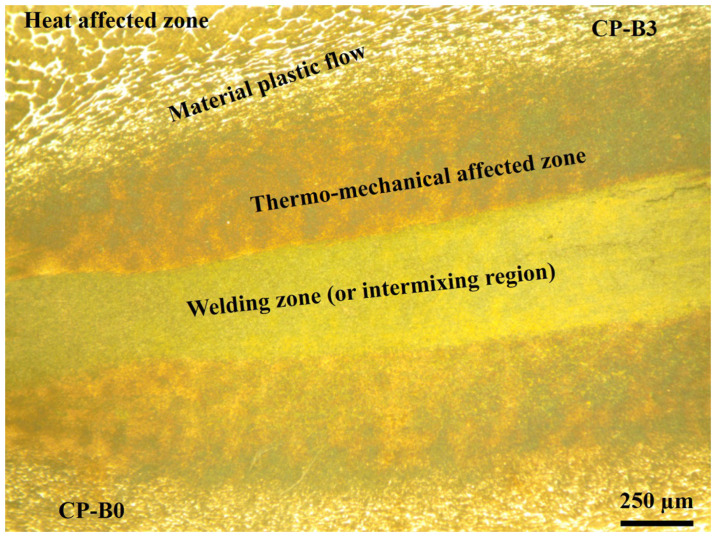
Microstructural details in the achieved metallic joint between the similar CP-B0/CP-B3 welded steels.

Figure 8 shows the microstructural features formed in the RFW of one of the studied similar advanced steels. The main challenge during these RFW tests was to control the thermomechanical processing, due to the very complex nature of the chemical composition of the advanced steels, considered in this work as highly alloyed steels, which, in addition to the obvious thermophysical differences, such as the melting point, may cause brittle components in the joint. Therefore, due to the intense thermomechanical treatment that the material undergoes, combined with the operating conditions of the RFW process, microstructural phenomena, such as dynamic recrystallization and grain refinement, solid-state phase transformations, deformation-induced precipitation, solid dissolution, and grain growth, can occur [16]. Consequently, the formation of the microstructure of the metallic joint and the corresponding mechanical resistance can be influenced (positively or not) by all of these metallurgical phenomena. It is therefore expected that, in these advanced welded steels, these phenomena are manifested in the welded zone.

**Figure 9:**
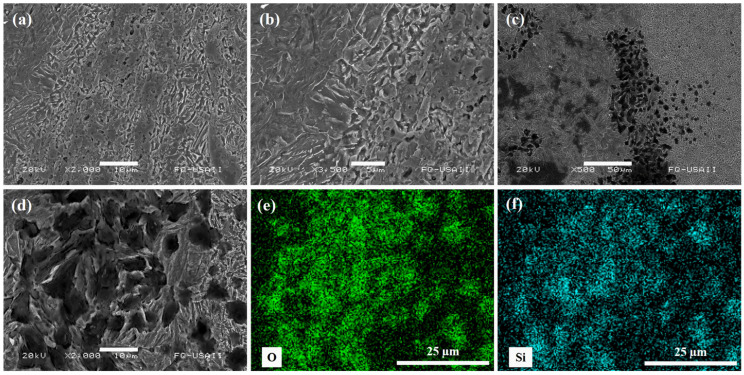
SEM micrographs for the similar Q&P-B0/Q&P-B3 welded steels: (**a**–**d**) continuous magnification in the welding zone and (**e**,**f**) elemental mapping for the oxygen and silicon in the same welding zone.

The SEM examination has allowed us to analyze the metallic joint between the advanced welded steels, as shown in Figure 9, for the similar Q&P steel weldments. The presence of some interlayers and particles in the intermixing region have been identified. The EDS analysis indicates that the particles principally correspond to SiO_2_ compounds. Based on these indications, their formation is mainly attributed to the oxidation process that takes place during the high-temperature RFW processing. Hence, the interaction of most affinity elements in the steel with the oxygen present in the atmosphere promotes this behavior. A similar behavior occurs in the dissimilar welds. This manifestation of oxidizing principal elements in the different steels can result in a process limitation when the welding process is not protected, such as in the case of using inert gas. Al-Moussawi and Smith [35] have categorized some defects in this type of welding that can also contain Fe, Mn, Si, Al, and O nonmetallic interlayers that are prompt to form in the intermixing region when applying the friction stirring welding technology in microalloyed hot-rolled steels. Also, microcracks can appear in this region, as happened in the dissimilarly welded steels, as shown in Figure 7a, which can be attributed to the hard particles formed or lodged in the intermixing region. Other types of defects correspond with the concentration of the deformation bands, observed in the same Figure 7a, for the case of the dissimilar welds. These bands can have a direct mechanical influence in the welded zone due to the redistribution of the element concentrations that result from the plastic flow material during the friction [36].

**Figure 10:**
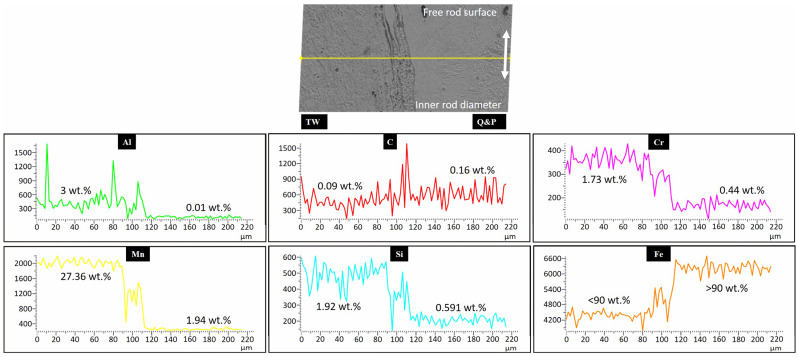
Concentration profile behavior of the principal alloying elements in the intermixing region in the dissimilar TW-B2/Q&P-B0 welded steels. Yellow line in the SEM image represents the data acquisition and white arrow indicates the direction to the inner diameter and free surface of the rod.

However, Figure 10 shows the EDS line scan analysis carried out across the welding interface in the dissimilar TW-B2/CP-B0 welded steels, which also presents a remaining fragment of the nonmixed material in the Q&P steel side. This SEM–EDS analysis has been obtained near the end of the rod diameter, with the aim of covering more certain information from a punctual area rather than in the middle of the rod, in which the welding zone is very large. This was decided with the objective of knowing with greater certainty the element redistribution in the intermixing region. From the information, it is clear that there is a distinct behavior of the alloying element concentration in the intermixing region. C and Al increase their content, while Cr follows a constant decreasing behavior from the TWIP steel side to the Q&P steel side. In the case of Mn and Si, they show the same intermediate concentration profile behavior from the TWIP steel side to the Q&P steel side. Fe behaves in the same way as Mn and Si, but in the opposite direction, from the Q&P steel side to the TWIP steel side. Knowing that the intermixing region has approximately a specific element concentration, it can be expected that this region will manifest different physicochemical properties than the base steels. This solute redistribution can occur during the thermomechanical processing under the viscoplastic condition of the surfaces being joined [37].

### 3.3. Microhardness Profile of the Welded Steels

**Figure 11:**
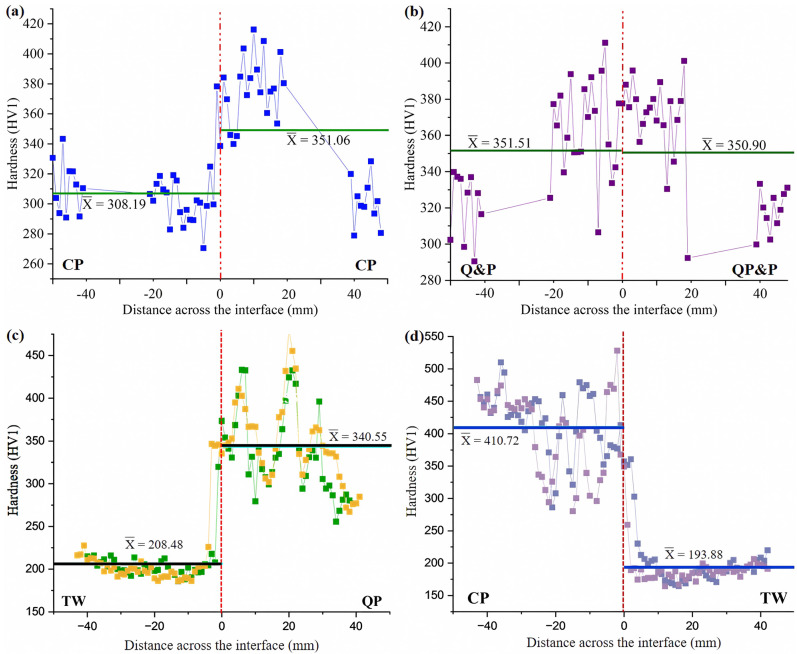
Vickers microhardness profile for the rotary friction welding tests: (**a**,**b**) similar welds and (**c**,**d**) dissimilar welds.

**Figure 12:**
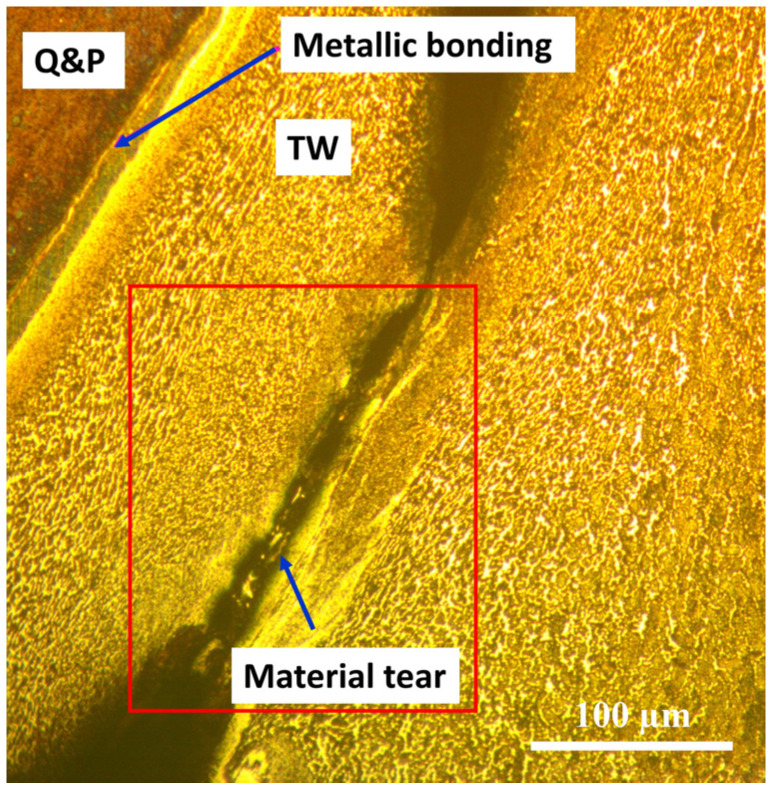
Optical micrograph of the dissimilar Q&P/TW welded steels showing the mechanical resistance of the metallic bonding instead of the material in the heat-affected zone by the RFW process.

Figure 11a–d shows the microhardness measurement profiles of the similarly and dissimilarly welded steels. It can be seen that, for the similar CP welds, the microhardness mean values are quite different, as shown by the effect that the boron element has in refining the grain [27]. And, in the case of the Q&P welds, the same behavior is observed due to the multiphasic microstructures in both steels. However, in the case of the dissimilar welds, there are differences between the mean hardness values. According to this, it is observed that the TWIP steels have the lower hardness, whilst the CP and Q&P steels have the higher ones; this means that the TWIP steel is the softer material. Hence, it can be inferred from these hardness values that the microstructure in each steel has a direct influence on this property. This behavior explains the fact of the flash shape for the dissimilar welds in Figure 5c,d. Other relevant information is that, in the soft steels around the welding interface, this zone tends to increase the hardness values as a consequence of the finer grain structure. In the specific case of the TWIP steels, it is reasonable to think that the mechanical twinning was eliminated by the effect of the thermomechanical processing and that the soft, coarser annealing twins have been created instead. In consequence, it can again be stated that the typical hardness profile (e.g., the bell-shaped curve) achieved by this RFW technique in most metals is not the same for the present results. Hardness fluctuations are reported elsewhere due the chemical composition and microstructure heterogeneities in the materials to be welded [38]. On the contrary, during the dissimilar welding tests, a macrocrack was generated which originated from the welding system being stuck, as shown in Figure 12. After the metallographic preparation of the welded sample, the revealed microstructure indicates that this type of welding really has the superior mechanical resistance at the interface line. This means that the metallic bonding was not affected or damaged, but the heat-affected zone was torn.

### 3.4. Corrosion Behavior of the Welded Steels

**Figure 13:**
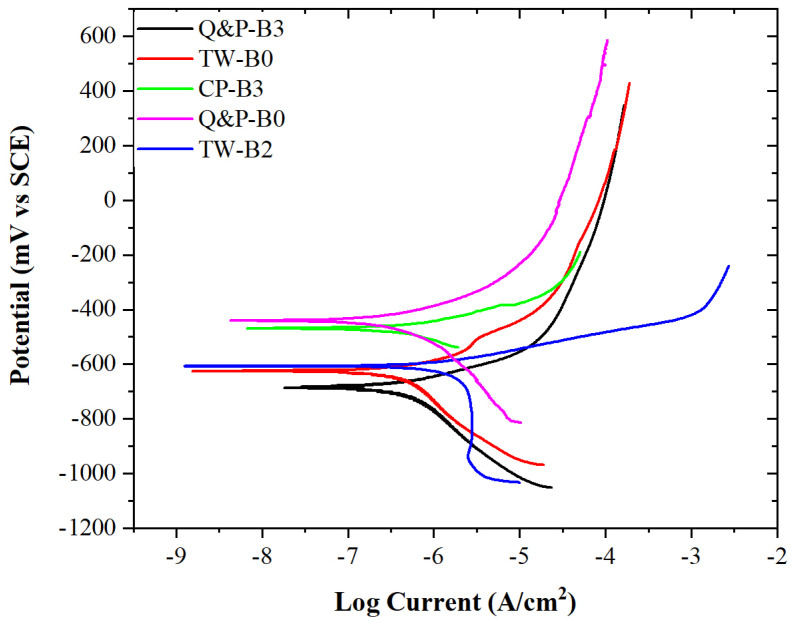
Potentiodynamic polarization curves of the differently welded advanced steel samples exposed to the 3.5% NaCl solution.

Figure 13 shows the potentiodynamic polarization curves obtained for the advanced-rotary-friction-welded steels using the lab-made microdroplet electrochemical cell. The plot shows the typical response acquired for the different steels for each measured point. It is observed that the composition of the steel modifies the measured corrosion potential (E_corr_) and corrosion current density (I_corr_). Likewise, two distinct zones, corresponding to the cathodic and anodic reactions, appear on the potentiodynamic polarization curves. Below the E_corr_, the cathodic reduction reaction is evidenced. This behavior is associated with the oxygen reduction reaction (O_2_ + 2H_2_O + 4e^−^ → 4OH^−^), which is the most common reaction carried out in chloride solutions and near-neutral pH media [39]. Additionally, a mixed activation–diffusion control is evidenced. Contrarily, when the potential becomes more positive than E_corr_, the metallic matrix begins to constantly dissolve. Since iron is the main constituent of steel, this behavior is mostly attributed to its dissolution (Fe → Fe^2+^ + 2e^−^). Also, it is important to mention that there is no evidence of a protective formation corrosion product layer, but an active dissolution behavior is observed. A limiting current density is reached as the potential becomes more positive. This behavior is related to the diffusion through the corrosion products layer [40].

**Figure 14:**
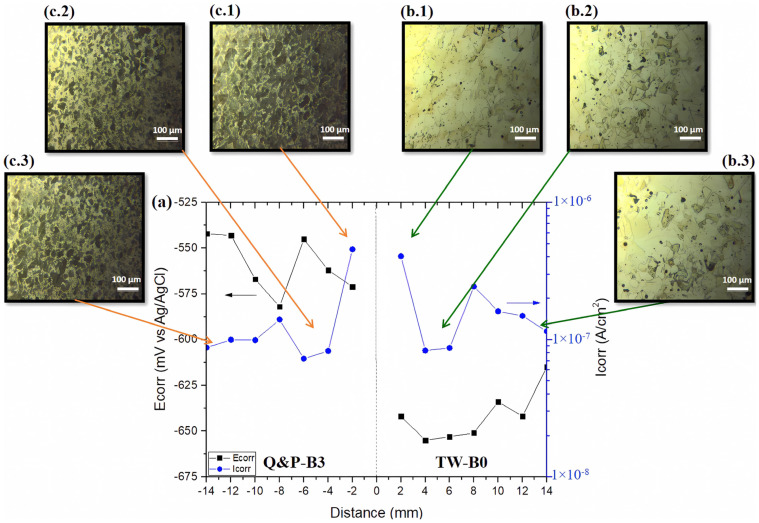
(**a**) Measured electrochemical parameters for the dissimilar TW-B0/QP-B3 welded steels, (**b.1**–**b.3**) the microstructures of the welded TWIP steel, and (**c.1**–**c.3**) the microstructures of the welded Q&P steel.

Figure 14 shows the measured parameters of the potentiodynamic polarization curves of the dissimilar QP-B3/TW-B0 welded steels. The plot in Figure 14a shows that the electrochemical behavior of the welded steels differs at each measured point. Also, it is observed that, near the interface of the welded TWIP steel, there are the more negative values of E_corr_ and the higher values of I_corr_ compared to the test points at −2 and −14 mm. These results suggest that the corrosion susceptibility of the welded TWIP steel increases from the heat-affected zone towards the weld zone. Then, this corrosion susceptibility tends to decrease as the length increases from the welding interface, which increases to its own side in the base zone. It is worth noting that the chemical composition remains unaffected during the RFW process because the lower working temperatures, as contrarily happens in other welding processes [41]. The change of the electrochemical behavior is attributed to the distortion of the microstructure in the welded TWIP steel due to the possible recovery of its microstructure [42]. Then, the microstructure is coarsened and there is the appearance of annealed twins, as can be seen in Figure 14b(1–3). During the thermomechanical process, the density of the slip planes increases, generating more electrochemically active surfaces as a consequence of the higher amount of stored energy in the grain substructure, which raises the galvanic corrosion effects in the microstructure. In the case of the welded Q&P steel, it shows a similar behavior as that manifested by the welded TWIP steel. So, the electrochemical results indicate a higher I_corr_ near the welding interface, which is diminished by one order of magnitude as the test point moves away from the joint interface. This behavior can be attributed to the plastic deformation achieved during the RFW process, as shown in Figure 14c(1–3).

**Figure 15:**
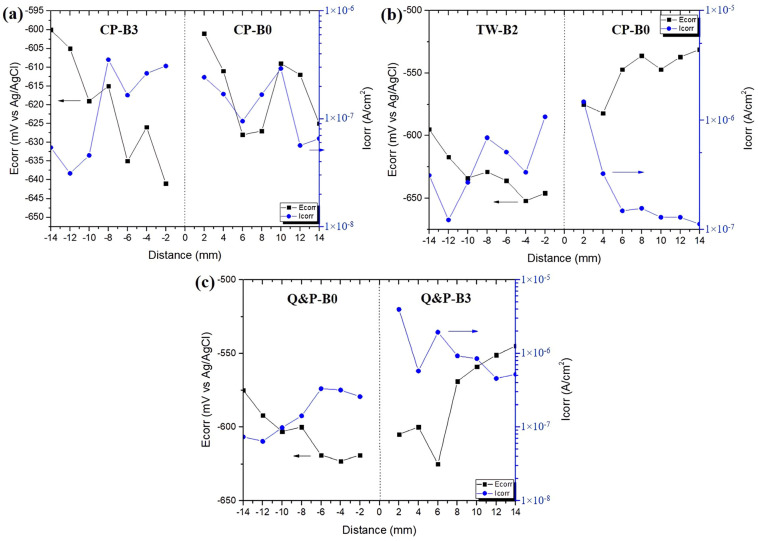
(**a**–**c**) Corrosion potential (E_corr_) and corrosion current density (I_corr_) values measured for the similarly and dissimilarly welded steels.

Figure 15a–c shows the measured parameters obtained for the similar CP-B0/CP-B3, CP-B0/TW-B2, and Q&P-B0/Q&P-B3 welded steels, respectively. The plots show a similar behavior as described above for the dissimilar welds, indicating that the RFW process promotes zones of higher activity prone to corrosion in the region near the welding interface, being associated to the higher distortion of the microstructures. This behavior remains constant regardless of whether the joints are similar or dissimilar.

## 4. Conclusions

Using the first, second, and third generations of advanced steels, rotary friction welding (RFW) tests were carried out as an effort to determine their processing feasibility under similar and dissimilar weld conditions. Thus, it was possible to achieve metallic bonding between joined steels under fixed RFW parameters (i.e., the rotation speed and forge loading). In accordance, it was observed that the welding zone widens in the center of the workpiece. Modification of the initial microstructure of the steels by the imposed thermomechanical processing has promoted that TWIP steels behave in softer ways than the CP and Q&P steels (TWIP steel has the lower hardness values, whilst CP and Q&P steels have the higher ones), in accordance with the microhardness measurements. Thus, the obtained microhardness profiles showed that the steels welded by the RFW process did not follow the typical bell-shaped curve. In the case of the dissimilar welds, the elementary SEM–EDS analysis revealed an intermediate solute concentration in the intermixing region dominated by the elements of the most-alloyed steel (i.e., TWIP steel). Furthermore, interlayers and some particles in the welding zone were found that can fragilize the metallic joint by the generation of defects, such as cracks. In addition, the electrochemical response of the welded steels showed that the compositional and microstructural condition mostly affect the corrosion trend. The similarly and dissimilarly welded steels also showed that the corrosion increases at the welding interface and decreases away from it. This corrosion behavior is mainly attributed to the energy that is stored in the distorted microstructure of each steel. Finally, from the present results, this RFW approach using high-alloyed advanced steels (i.e., AHSSs) represents a different way of applying these steels in the automotive industry. Obviously, more work needs to be carried out to clarify in-depth the metallurgical changes that occur when welding the steels under similar and dissimilar conditions by varying the welding parameters.

## Figures and Tables

**Table 1:** Chemical composition of advanced high-strength steels used for the RFW tests (in wt.%).

Element (wt.%)	CP-B0 Steel	CP-B3 Steel	TW-B0 Steel	TW-B2 Steel	Q&P-B0/P2 Steel	Q&P-B3/P2 Steel
C	0.16	0.16	0.09	0.09	0.16	0.16
Mn	1.94	1.93	27.36	27.36	1.94	1.93
Si	0.591	0.59	1.92	1.92	0.591	0.59
Al	0.012	0.01	3.0	3.0	0.012	0.01
Cr	0.44	0.44	1.73	1.73	0.44	0.44
Ni	0.12	0.12	0.85	0.85	0.12	0.12
Mo	0.401	0.397	0.29	0.29	0.401	0.397
Cu	0.068	0.068	0.2	0.2	0.068	0.068
Ti	0.018	0.014	0.03	0.03	0.018	0.014
Nb	0.151	0.156	0.0	0.0	0.151	0.156
V	0.01	0.01	0.0	0.0	0.01	0.01
B	0.0	0.006	0.0	0.047	0.0	0.006
S	0.031	0.03	0.02	0.02	0.031	0.03
N	0.012	0.012	0.012	0.012	0.012	0.012
Fe	Bal.	Bal.	Bal.	Bal.	Bal.	Bal.

## Data Availability

The authors declare that the data supporting the findings of this study are available within the paper.
